# LAITOR - Literature Assistant for Identification of Terms co-Occurrences and Relationships

**DOI:** 10.1186/1471-2105-11-70

**Published:** 2010-02-01

**Authors:** Adriano Barbosa-Silva, Theodoros G Soldatos, Ivan LF Magalhães, Georgios A Pavlopoulos, Jean-Fred Fontaine, Miguel A Andrade-Navarro, Reinhard Schneider, J Miguel Ortega

**Affiliations:** 1 Computational Biology and Data Mining Group, Max-Delbrück Center for Molecular Medicine. Robert-Rössle-Strasse. 10, D-13125, Berlin, Germany; 2 Laboratório de Biodados, Dpto. de Bioquímica e Imunologia, ICB - UFMG. 31270-901, Belo Horizonte - MG, Brazil; 3 European Molecular Biology Laboratory, EMBL-Heidelberg, Meyerhofstrasse 1, 69117, Heidelberg, Germany; 4 LIFE Biosystems GmbH. Poststrasse 34, D-69115, Heidelberg, Germany

## Abstract

**Background:**

Biological knowledge is represented in scientific literature that often describes the function of genes/proteins (bioentities) in terms of their interactions (biointeractions). Such bioentities are often related to biological concepts of interest that are specific of a determined research field. Therefore, the study of the current literature about a selected topic deposited in public databases, facilitates the generation of novel hypotheses associating a set of bioentities to a common context.

**Results:**

We created a text mining system (LAITOR: ***L**iterature **A**ssistant for **I**dentification of **T**erms co-**O**ccurrences and **R**elationships*) that analyses co-occurrences of bioentities, biointeractions, and other biological terms in MEDLINE abstracts. The method accounts for the position of the co-occurring terms within sentences or abstracts. The system detected abstracts mentioning protein-protein interactions in a standard test (BioCreative II IAS test data) with a precision of 0.82-0.89 and a recall of 0.48-0.70. We illustrate the application of LAITOR to the detection of plant response genes in a dataset of 1000 abstracts relevant to the topic.

**Conclusions:**

Text mining tools combining the extraction of interacting bioentities and biological concepts with network displays can be helpful in developing reasonable hypotheses in different scientific backgrounds.

## Background

The richness of information generated by different research groups is sometimes focused on issues that lack explicit connection with those generated by colleagues from other groups. However, currently, there are available literature mining techniques that permit to connect the knowledge generated by distinct groups and improve the understanding of some key points of their research [1]. Text mining machines have been created to mine the biological information in a trial to establish new biological concepts from previous knowledge [2-4]. These machines were proven to be reliable in extracting biological facts either analyzing full text [5,6] or just condensed information present in the abstracts of scientific papers [7,8] as stored in the MEDLINE database.

Text mining techniques for information-retrieval comprise some basic steps: to find relevant articles in the research field of interest; to identify the biological entities cited in the text, as well as to disambiguate confuse bioentity names (i.e. genes and proteins) within and among distinct species; to infer putative relationships between bioentities based on co-occurrence of biological terms in the same article, abstract, sentence or phrase [2]. Recently, AliBaba has been developed to graphically visualize information on associations between biological entities extracted from PubMed using pattern matching and co-occurrence filtering (http://alibaba.informatik.hu-berlin.de/, [9]). Later, a system called NetSynthesis [10] has been developed to permit the controlled building of biomolecular networks by users, where the searching criteria on PubMed are customized by using parse tree query language [11]. However, these systems do not permit the integration of customized dictionaries on their algorithm.

We present here a system called LAITOR (***L**iterature **A**ssistant for **I**dentification of **T**erms co-**O**ccurrences and **R**elationships*). This software was developed to normalize the bioentities names tagged in the abstracts to a user defined protein dictionary; as well as to extract their co-occurrence, along with other protein or important biotic/abiotic stimuli terms, the later implemented as a customized concept dictionary. Such co-occurrences are extracted taking into consideration the presence of terms in the same sentence of scientific abstracts and adopting a set of rules to filter bioentity pairs that occur in several sentence structures (see details in Implementation). The software performed as a greatly precise method. Here, it has been used to mine protein co-occurrences related to green plant-pathogen interactions.

## Implementation

### Abstracts retrieval

In order to retrieve scientific abstracts related to green plants that would be related to defense mechanisms, we have used the system MedlineRanker [12]. Two MeSH http://www.nlm.nih.gov/mesh/ terms (Host-Pathogen Interactions AND Plants) have been used as "training dataset" to rank 10,000 recently-published abstract from the whole MEDLINE database. After the MedlineRanker analysis we retrieved the top 1,000 PubMed IDs from the generated rank to be loaded as "application dataset" for the next steps of our analysis [Additional file 1].

### Protein tagging

LAITOR is optimized to work by analyzing tagged scientific abstracts. For this purpose, we adopted the NLPROT [13] program as LAITOR's protein tagger. The plain text format (-f txt) must be chosen for the NLPROT output file, where bioentity names present in the text are tagged between "<n>" and "</n>" tags. The tagged protein names are filtered according to a user-defined bioentity dictionary, in our case study: a plant protein name and synonym dictionary.

### Protein Dictionaries

Two protein dictionaries have been generated for the development of LAITOR. The first (named human proteins dictionary) created for the evaluation of LAITOR performance (explained below) in the BioCreative II Interaction Article Subtask (IAS) [14]. The second (named plant protein dictionary) has been used in the identification of co-occurring of green-plant protein pairs retrieved for abstracts related to host-pathogen interactions.

The human protein dictionary has been created by using all the protein records deposited for *Homo sapiens *[NCBI Taxonomy id: 9606] in the UniProt-SwissProt-TrEMBL (UP-SP-TR) database. In this dictionary, the definition(s) and synonym(s) for all human UP-SP-TR proteins are included. Furthermore, for each record, the corresponding NCBI Gene symbol and synonyms were used to enrich the representative terms of said protein. At the end, the human proteins dictionary is composed by 87,537 records (IDs), comprising a total of 112,686 distinct protein terms, which have been completed by the addition of 40,234 supplementary terms from the NCBI Gene database.

Additionally, specific genes names and synonyms for every organism deposited in the NCBI Taxonomy database that have gene records in the NCBI Gene database have been used to create LAITOR readable dictionaries. To use these dictionaries, users must inform the taxonomy identification number (Taxonomy ID) for the preferred organism followed by the extension ".dictionary" (e.g. "9606.dictionary" for "Homo sapiens" genes) during set up, as explained at LAITOR's documentation file.

For the plant dictionary, the complete Gene tab-delimited database from Entrez website has been downloaded (5,317,958 records), which comprises 505,403 different organisms (Taxonomy IDs - TAXIDs). To filter only those records related to green-plant proteins, we used the NCBI Taxonomy database to select from the Gene table only those records with a TAXID corresponding to Viridiplantae organisms, which included 99,488 different records. At the end, the plant protein dictionary contained 148 plants organisms (0.02% of total organisms) and a total of 237,077 Gene records (4.45%), which included 217,224 distinct protein symbols and 62,521 synonyms (see one example for the Gene PR1 of *Arabidopsis thaliana *[GenBank: 815949] in Additional file 2).

The resulting table displays two columns: one for the bioentity names, and the second with their respective synonyms so that it can exist as lines (records) as synonyms for each bioentity name (Additional file 2).

### Name ambiguity

Another aspect explored by LAITOR, is how to handle gene name ambiguity. The strategy of using the Taxonomy database to limit the number of used entries reduced the possibility of inclusion of names of other organisms which would cause ambiguity among terms. However there are terms that commonly occur for more than one organism, or different proteins from the same organism that share the same name or synonym. To cope with this, LAITOR creates a tag file in which the ambiguous terms identified in the analysis are normalized to the same name in the protein dictionary. Such terms that match multiple protein names or that are synonyms of multiple protein names are marked in the LAITOR output. This warns users about the possibility of misinterpretation for such a term.

### Concepts Dictionary

In order to check the co-occurrence and likely involvement of plant proteins names along with biotic and abiotic stimuli names, a list of previously known stimuli and their synonyms has been provided as Concept Dictionary (for example: Jasmonic Acid, Jasmonate and JA were included as the same concept). Both, Protein and Concept Dictionaries are available as additional material [Additional files 3 and 4].

Additionally, in order to attend different contexts, we have populated all the sub-headings of NCBI's Medical Sub Headings (MeSH) Trees (available at http://www.nlm.nih.gov/mesh/trees.html) as LAITOR's concepts dictionaries, as explained at LAITOR's documentation.

### Biointeractions Dictionary

A list representing the different types of interactions or relationships between proteins was generated based on previously published list [4,15]. It is composed by 76 terms, which have been included together with a total of 886 synonyms as seen in Additional file 5, Table S2. Considering all terms, the biointeraction dictionary in its entirety is composed of 963 different words.

### Co-occurrence analysis

Once the abstracts to be analyzed had been retrieved and tagged for protein and gene names, biointeractions and concepts, LAITOR was used to perform a co-occurrence analysis [see Additional file 6].

At the sentence level, each line of the tagged abstracts was divided at every full stop (".") punctuation sign. We paid special attention to the presence of these full stop marks in alternative positions that did not indicate the end of the period, as in the case of species names (for example: *A. thaliana*) or protein names (for example: PDF1.2 protein).

Initially the whole abstract is screened to store the occurrence of all bioentity names. After storage of all names, each protein name is checked for its occurrence in each of the separated sentences. If a bioentity term is found, let us name this term as "Pair 1", the script checks the occurrence of a second bioentity name, "Pair 2", different from Pair 1 in the same sentence. To avoid redundancy, the script checks on-the-fly if Pair 2 is a synonym of the previously identified Pair 1 and discards such cases.

It has been previously published that 90% of the bio-interactions among proteins documented in the literature adopts the pattern "Protein-Biointeraction-Protein" [16], this pattern being chosen by approaches like iHOP [15] and HomoMINT [17]. Nevertheless, we adjusted LAITOR to identify other patterns of Protein-Protein or Protein-Concept co-occurrence, as explained below.

The co-occurrences identified by LAITOR are classified into four types. From the most to the least stringent, these types are:

**Type 1**: Both co-occurring protein names/synonyms must not refer to the same protein (common for all types of co-occurrences), they must be present in the same sentence of the abstract and, additionally, it is required that a term from the Biointeractions Dictionary occurs in between the considered terms. An extra optional step is the identification of a biological stimuli (represented as a term from the Concepts Dictionary) term anywhere in the sentence, which is then associated to the interacting pair;

**Type 2**: Same as Type 1, except that the biointeraction may occur anywhere in the sentence;

**Type 3**: Same as Type 1, except that the occurrence of a biological term in the sentence is not required;

**Type 4**: All the pairs of co-occurring protein names/synonyms mentioned in the abstract are considered, whether they are in the same sentence or not.

Thus, when LAITOR performs under type 4, the other co-occurrence types are included.

Multiple co-occurrences of type 1, 2 and 3, might happen in a given sentence. To cope with this, our system was adapted to perform an overlapped search. This means that in cases where two proteins (A and B) occur along with the same biointeraction, like in the sentence "A and B regulate C", the pairs "A-regulate-C" and "B-regulate-C" are identified as type 1 co-occurrences. Note that the co-occurring pair "A-B" will be assigned type 2. Moreover, in more complex sentences such as "A is regulated by B and activates C", the system will retrieve as co-occurrences of type 1 "A-regulated-B", "A-regulated, activates-C', and "B-activates-C" (together with type 2 "A-regulated, activates-B" and type 2 A-regulated, activates-C) thus over predicting the number of different bio-interactions between the A, B and C proteins. However such complex sentences may not be very frequent. In order to determine if they are a serious problem, we performed a series of manual evaluations of the results of LAITOR's analysis on several abstract datasets.

### Performance evaluation

Protein term co-occurrences at sentence level of scientific abstracts might be potentially useful for the prediction of literature-based protein-protein interactions. Therefore, we have tested the performance of LAITOR to find protein-protein interaction data in abstracts. For this purpose, we have used the BioCreative II test dataset for the Interaction Article Subtask (IAS) as gold standard [14]. This "performance evaluation dataset" is composed of relevant (3,529) and irrelevant (1,957) abstracts for the curation of protein-protein interactions present in the MINT and IntAct databases [18]. Once LAITOR identifies a co-occurring protein pair in an abstract, this is considered to be positively (relevant) classified. After the classification of all gold standard abstracts the precision and recall are calculated for each of the four co-occurrence types (1-4), and the performance compared to methods participating in the BioCreative II challenge. A receiver operating curve (ROC) was created by using the package ROCR [19]. Positive and negative performance evaluation datasets are provided as additional material [Additional file 7].

### Network representation

A protein and stimuli co-occurrence analysis created by LAITOR from PubMed abstracts is parsed from a general output file into a tab-delimited text file (extension .co) that is used as input by most network visualization software. As default, LAITOR generate inputs for two of these programs: EMBL Medusa [20] and EMBL Arena3D [21], which provide networks in one- and multi-dimensional charts, respectively, enabling the complex output generated by LAITOR to be efficiently handled.

## Results and Discussion

### LAITOR's developmental pipeline

**Figure 1:**
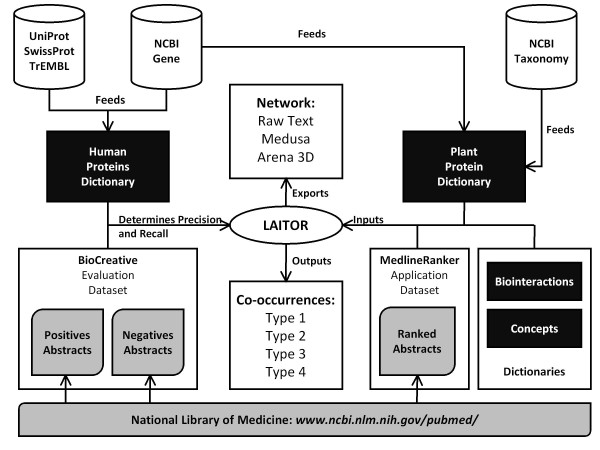
**Pipeline for LAITOR's development**. LAITOR's has been evaluated for correct classification of abstracts relevant to curation of protein-protein interactions from BioCreative II challenge (evaluation dataset). Co-occurrences of terms from the human proteins dictionary in these abstracts have been used as an indicator of relevance. Precision and recall have been measured as 0.89 and 0.48 respectively for the type 1 of co-occurrences. Afterwards, for abstracts ranked to be related to host-pathogen interactions in plants (application dataset), LAITOR has generated a list of co-occurrences and a network representation of the terms from the plant protein dictionary which could be found in this dataset. **Symbol key**: dark rectangles: dictionaries; grey shapes: abstract datasets; cylinders: public databases; ellipse: LAITOR script; white rectangles: LAITOR's outputs;

LAITOR has been developed by combining a flexible rule-based method together with a pre-defined vocabulary match approach. Figure 1 illustrates the pipeline for LAITOR's development, which is explained in detail in the following sections.

LAITOR uses as input a set of scientific abstracts as stored in the records of the MEDLINE database. Abstracts are analyzed individually for co-occurrences, which are extracted and classified into four types according to the rules described in Implementation section. Additional file 8, Figure S1 exemplifies a tagged sentence extracted from the PubMed article identified by PMID 19061405. The co-occurrence analysis starts by (i) the creation of a list with the occurring bioentities (proteins or genes, [see Additional file 2, Table S2]) and stimuli names present in precompiled dictionaries (see Implementation), for the whole abstract. In the example the names detected were: HSP90, RAR1 and SGT1. (ii) Further, each sentence is queried for the co-occurrences of different bioentity names establishing pairs. In this example the co-occurrences of the types 1, 2 and 3 are defined as follows.

Type 1: the pairs HSP90 and RAR1, as well as, HSP90 and SGT1 were both extracted with the interleaved biointeraction term "interact" associating the members of each pair (see Additional file 5, Table S2 for example of a Biointeraction term representation in the Biointeraction Dictionary).

Type 2: the pair RAR1 and SGT1 was extracted, with the occurrence of the biointeraction "interact" in the same sentence, however not interleaved.

Type 3: Other co-occurrences of the protein terms (HSP90, RAR1 and SGT1) found in the same sentence were considered as co-occurrences of type 3.

Furthermore, the combinations of all the bioentity names identified in the abstract, except synonyms, are considered as co-occurrences of type 4 (see Implementation for explanation).

### Evaluation against BioCreative II

LAITOR was compared to the Interaction Article Subtask (IAS) of BioCreative II text mining challenge [14]. Table 1 shows that LAITOR could predict abstracts considered relevant for the curation of protein-protein interaction (evaluation dataset) with a maximum precision of 0.89 and a corresponding recall of 0.48 considering type 1 co-occurrences (bioentities co-occur within the same sentence, and they are interleaved by some biointeraction term; see Implementation for a detailed description). Among the 19 evaluated methods for the IAS task, LAITOR's predictions (considered to be a non SVM-based prediction) demonstrated to be the second most precise method keeping a reasonable sensitivity (recall) index. In predictions using the co-occurrence types 2-4, which do not require the presence of a biointeraction term, LAITOR produced results with a precision ranging from 0.82 to 0.85, a recall ranging from 0.61 to 0.70 and a F-score ranging from 0.60 to 0.72 (See Table 1 for values for each type). This implies that LAITOR's detection of protein co-occurrences with biointeraction terms improves precision that the expense of a small reduction of recall and therefore increases the likelihood of filtered protein pairs from such abstracts will indeed display biologically relevant fact.

**Table 1:** LAITOR evaluation against BioCreative II IAS subtask.

Type	Precision	Recall	F-score	Accuracy
1	0,89	0,48	0,63	0,63

2	0,85	0,61	0,71	0,68

3	0,83	0,62	0,72	0,68

4	0,81	0,70	0,60	0,60

Manual examination of some false-positive abstracts showed that although the biointeraction was not correctly identified, the selected sentences described a relevant biological interaction. For example, this sentence: "*Taken together, these results suggest that loss of RPA1 activates the Chk2 signaling pathway in an ATM-dependent manner*" (PMID: 15620706), was interpreted as RPA1 activates Chk2 because the term "activates" was found between the protein names RPA [Entrez Gene id: 6117] and Chk2 [Entrez Gene id: 11200]. The sentence actually indicates a different relation but it is informative in terms defining a functional relation between these two proteins.

**Figure 2:**
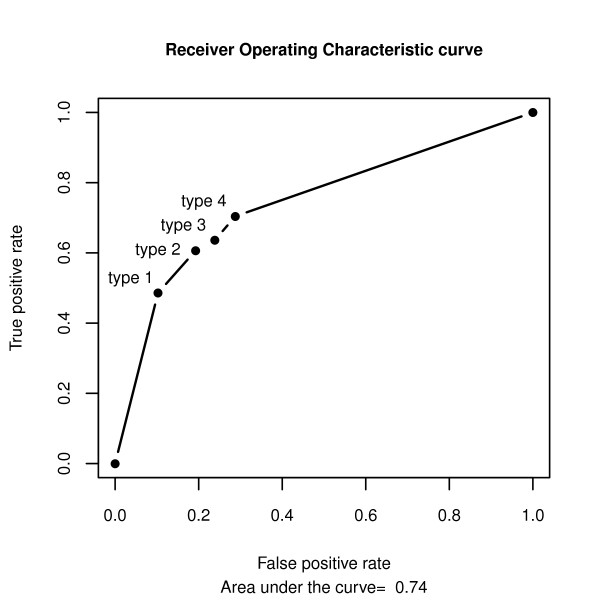
**Receiver Operating Characteristic (ROC) curve for LAITOR predictions**. The corresponding area under the curve (AROC) is 0.74, calculated using the four types of interactions found in such an abstract as a measurement of its overall predictive power. Note that higher types include the lowest ones.

In further comparison of LAITOR's performance with other methods from the BioCreative II challenge in order to correctly classify the IAS gold standard abstracts, we scored LAITOR's prediction of these abstracts with a score S = 5-T where T, that is the type of co-occurrence, ranges from 1 to 4, according to the presence of at least one sentence displaying a co-occurrence of types 1 to 4 (adopting S = 0 when no co-occurrence is detected in the abstract). Then, we calculated the area under the receiver operating curve (AROC), corresponding to 0.74 (Figure 2).

### Case-study: co-occurrence analysis of terms related to a plant-pathogen interaction dataset

We performed a case study by applying LAITOR to generate a list of green plant's protein co-occurrences related to host-pathogen interactions. Plants respond to diverse environmental stimuli, biotic and abiotic, by mobilizing specific protein networks used to identify its source and to activate the cellular mechanisms to surpass changes caused by stressful conditions. Commonly, the adaptative responses found in plants are flexible and the same subset of proteins/genes can be activated by different types of stimuli, including defense against pathogens or tolerance under severe environmental conditions [22]. Therefore, a system like LAITOR used in this context should be expected to be useful in suggesting novel roles for known protein interactions.

Moreover, this topic is important for plant biotechnological and physiological studies, since (i) diverse economically important crops are attacked by several phytopathogens in the field, which is prejudicial for agricultural practices along the world [23], and (ii) cultivated lands are often affected, for instance, by severe abiotic conditions such as high salinity [24], drought [25], over-flooding [26] or extreme cold [27]. As a result of this interest, during the last few decades several efforts have been dedicated to characterize these mechanisms, which resulted in a fair amount of related publications deposited in MEDLINE. These data comprises proteins or entire protein networks that are used by plants, as well as chemicals identified to have a key role in the signaling pathways that establish the plant adaptative responses. Jasmonic acid (JA) [28], ethylene (ET) [29] and salicylic acid (SA) [30,31] are examples of phytohormones employed by plants that act as signaling molecules in diverse defense response networks [32]. This wealth of data facilitates a text mining procedure such as LAITOR.

A total of 1,000 abstracts on the topic of green plant's host-pathogen interactions were retrieved with MedlineRanker [26] (application dataset) and analysed with LAITOR, of which 79 displayed at least one filtered co-occurrence. From the total 9,823 parsed sentences (including titles), 116 provided co-occurrences of the different types and pairs of bioentities (Table 2). A total of 263 pairs were retrieved from the application dataset.

**Table 2:** Survey of sentences and pairs extraction using the LAITOR algorithm on application dataset.

Type	Sentences	Pairs
1	25	52

2	35	66

3	24	27

4	N. A.*	21

**Total**	**116**	**263**

*N.A.: not applicable, as LAITOR does not consider sentences to extract co-occurrences of type 4.

In this dataset, a total of 68 different biointeraction terms could be identified among the co-occurring pairs, considering that the co-occurrences of type 3 do not restrict the filtering of biointeraction terms in the sentences. The top 10 most-common biointeraction terms and their frequencies within the application dataset are shown in Additional file 9, Table S3.

### Network visualization

LAITOR generates a network file relating the co-occurrences extracted. The nodes represent bioentities and the edges their co-occurrences in the set of abstracts used as input. Each edge is annotated by the type of co-occurrence from strictest (type 1) to least strict (type 4).

As an example we generated a network for a total of 51 nodes and 143 edges found in the application dataset only representing the co-occurrences of type 1, in order to reduce the complexity of the network [Additional file 10, Figure S2]. We illustrate the relevance for the analysis of using the dictionary of concepts in Additional file 11, Figure S3. It can be noticed that the displayed sub-network with 9 proteins (Additional file 11, Figure S3A; this is one of the subnetworks of the network represented in Additional file 10, Figure S2) gained two more members (catalase and SOD) when the concepts "oxidative stress" and "jasmonic acid" were also considered [see Additional file 11, Figure S3B]. The top 10 most-common terms present in the concept dictionary and their observed frequencies within the application dataset are shown in Additional file 12, Table S4.

### Hypothesis generation example

One of the most interesting applications of a co-occurrence based text mining analysis is the support given to new hypothesis generation [33,34]. Here we explore this functionality in LAITOR by examining the involvement of a common member of the photosystem response and disease signaling in *Arabidopsis *[see Additional file 13, Figure S4].

Accessing the abstracts analyzed by LAITOR and listed in Additional file 13, Figure S4B we observe that the *Arabidopsis thaliana *gene *RPS4 *(RESISTANT TO *P. SYRINGAE *4 [Entrez GeneID: 834561]) confers resistance to the bacterial pathogen *Pseudomonas syringae *carrying the avirulence gene *avrRps4 *[Entrez GeneID: 3555344, PMID: 8589423]. We can use LAITOR to find genes that could be hypothetically involved in resistance mechanisms regulated by *RPS4*. LAITOR associates this gene to several other genes. In the topic of resistance against pathogens *EDS1 *stands out: we can see that RPS4 requires the gene *EDS1 *(ENHANCED DISEASE SUSCEPTIBILITY1 [Entrez GeneID: 823964]) to confer *avrRps4*-independent resistance in tomato plants transiently expressing *RPS4 *[PMID: 15447648]. Using LAITOR we can see that there is another pathogen resistance gene that, similarly to *RPS4*, also requires EDS1, although in a different context [see Additional file 13, Figure S4A]. This is *PAD4 *(PHYTOALEXIN DEFICIENT4 [Entrez GeneID: 824408]), which confers resistance against the phloem-feeding green peach aphid (GPA) infesting *Arabidopsis*, and also requires its signaling and stabilizing partner *EDS1 *[PMID: 17725549].

Now, LAITOR shows that *PAD4 *is related to three genes: *LSD1 *[Entrez GeneID: 827786], *SIZ1 *[Entrez GeneID: 836163], and *WIN3 *[Entrez GeneID: 831173]. In more detail, a win3-T *Arabidopsis *(*WIN3*) mutant shows greatly reduced resistance to the bacterial pathogen *Pseudomonas syringae *carrying the avirulence gene *avrRpt2 *and expression of this gene at an infection site partially requires *PAD4 *[PMID:17918621]. The small Ubiquitin-like Modifier E3 Ligase (encoded by the gene *SIZ1*) interacts epistatically with PAD4 to regulate pathogenesis related gene expression and disease resistance [PMID: 17163880]. Finally, the disease resistance signaling components EDS1 and PAD4 are essential regulators of the cell death pathway controlled by LSD1 in *Arabidopsis *[PMID: 11595797].

Given the fact that both RPS4 and PAD4 require EDS1, one could explore weather or not these three known targets of PAD4 (SIZ1, WIN3, LSD1) could also be targets of RPS4, a fact not represented in the literature as evidenced by the absence of matches for the PubMed query "RPS4 AND (SIZ1 OR WIN3 OR LSD1)" [see Additional file 13, Figure S4C]. This example highlights the potential of LAITOR to unearth undiscovered public knowledge [35] using the condensed information of abstracts [36]. Thus, the system is able to extract precise information from the sentences in abstracts that can be used to generate new hypotheses.

### Current limitations of LAITOR

The main limitations of the system can be classified as those producing false positives and those producing false negatives co-occurring pairs. False negatives are mainly due to terms not recognized to be gene/protein names, and to failure to recognize a biointeraction. The first problem can be solved by improving the tagging mechanism and the underlying dictionaries. We approach the second by manually adding to the dictionary of biointeractions those that we find to be common. Some false positives co-occurrences are caused due to misrecognition of gene/protein names and/or biointeractions. The current tagging is conservative and therefore does not increase false identification of gene/protein names (see Material and Methods); it actually constitutes the slower step of the method. This ensures that the identified biointeractions actually point to relevant sentences. Most falsely identified biointeractions were originating from sentences with large numbers of genes. We are considering adding an option to dismiss sentences with more than two gene/proteins as a choice for users requiring greater accuracy.

### Comparison to other similar systems specialized in co-occurrence extraction

LAITOR is, as far as we know, the only method of co-occurrence detection along with customized that has been designed as standalone software to be included as part of other systems. However, LAITOR has some methodological particularities that merit comparison to recently developed systems that apply biological term co-occurrence as part of their functionalities.

STRING [37] is a web resource focused on a pre-compiled list of protein-protein interactions extracted by different methods. STRING uses Natural Language Processing [38] to search for statistically relevant co-occurrences of gene names, and also extract a subset of semantically specified interactions. Similarly, iHOP [15] is focused on the navigation of the scientific literature using biological term co-occurrence networks as a natural way of accessing PubMed abstracts. iHOP's text mining approach retrieves and ranks all the sentences for a given gene according to significance, impact factor of published journal, publication date or syntax structures where the gene occurs (i.e. gene-biointeraction-gene pattern). Furthermore, iHOP uses MeSH terms as source for information about gene function, what could be comparable to LAITOR's concepts search. Similarly to iHOP, co-occurrence methods have been developed for plant-directed literature analysis using *Arabidopsis thaliana *as a model [39]. This system, called PLAN2L, also classifies the extracted terms and co-occurrences as being related to physical and regulatory events for developmental processes, as well as with sub-cellular context, for that PLAN2L uses from co-occurrence to syntactic/semantic rule-based algorithms and supervised machine learning methods.

Although being designed for different purposes, we compared the features among LAITOR, STRING and iHOP (Table 3), once that these systems use biological term co-occurrences as part of their text mining strategies.

**Table 3:** Comparison of features between LAITOR, STRING and iHOP.

Features	LAITOR	STRING	iHOP
Software type	Command-line script	Website application	Website application

Information sources	Any type of text loaded by the user (e.g. PubMed, OMIM, Wikipedia)	PubMed, SGD, OMIM, The Interactive Fly.	PubMed

Text limit	Any type of tagged text	Only abstracts	Only abstracts

Protein name tagging	Depends of external software (NLPROT), confers against loaded dictionary	YES, filtered by selected organism	YES, filtered by selected organism

List of used synonyms	Flexible user-based dictionary input	Variety of pre-compiled dictionaries	Entrez Gene, FlyBase, UniProt and HUGO Nomenclature Committee

Explores biological concepts	YES, finds user loaded concepts linked to a co-occurring pair at sentence level.	NOT	YES, searches species names, MeSH and compound terms

Extracts co-occurrences among proteins	YES, considering whole text and isolated sentences	YES, limited to the whole abstract	YES, at sentence level only

Extracts interactions among proteins	YES, considering a biointeractions dictionary defined by the user	NOT	YES, considering a pre-compiled biointeractions dictionary

Terms co-occurrences	YES, extracts terms mentioned in the full text or in isolated sentences at different structures which are scored differently	YES, extract terms mentioned together in abstracts, more often than what would be expected by chance based on their overall occurrence	YES, extracts terms mentioned in isolated sentences

Semantic understanding	YES, extracts the biointeractions and concepts linked to an extracted pair at sentence level in different co-occurrence types	NOT, only checks co-occurrences of terms	YES, extracts the biointeractions and concepts linked to an extracted pair at sentence level

Co-occurrence frequency report	YES, displays the frequency that a pair co-occurred in general sentences, and for each found biointeraction	YES, only the number of times that a pair co-occurred in each abstract	NOT

Outputs network	YES, in tabular format and in pre-compiled formats for third-part applications (ARENA3D, MEDUSA)	YES, displays the network in the browser from selected abstracts	YES, users can build a network by adding a set of nodes per time by selecting desired abstracts

The main novelty of LAITOR in comparison to previous published software, besides the implementation of the concepts search, is the possibility to customize the dictionaries to be considered in the co-occurrence analysis (bioentities and biointeractions).

Reflecting this flexibility, we have included in the current LAITOR's distribution package a set of genes symbols/synonyms dictionaries pre-compiled from GeneDB records and divided by all the organisms deposited NCBI's Taxonomy Database http://www.ncbi.nlm.nih.gov/Taxonomy, in addition to the green plants dictionary used in the test case described above, making it possible to use LAITOR virtually for any species with gene data. Furthermore, in order to provide users with a wide set of relevant dictionaries for the concepts search, we compiled LAITOR's concepts dictionaries for each of the NCBI's Medical Subject Headlines (MeSH) main tree structures http://www.nlm.nih.gov/mesh/trees2008.html. The information about how to use these dictionaries is available in the documentation file of LAITOR.

## Conclusions

We presented here a new text mining software component called LAITOR, which performs co-occurrence analysis of scientific abstracts where biological entities are filtered from the tagged text using a user defined bioentity dictionary as support. Subsequently, a rule based system is used to detect the co-occurrence of such names along with biointeraction and, optionally, other biological terms provided by the Concepts Dictionary (such as stimuli), in scientific abstracts. We provide here an example of knowledge discovery by applying LAITOR to a subset of abstracts published about defense mechanisms in *Arabidopsis*. In this example, genes from different contexts (light and pathogen responses) have been placed together. Additionally, we have explored a new feature in biological text mining, which is the application of a user pre-defined concept dictionary in order to mine the literature and gather facts previously not reported together. Here, we have evidenced that the inclusion of the concept "oxidative stress" in the analysis conducted for *Arabidopsis *abstracts has brought two new members to a predicted gene network thought to be related to "jasmonic acid" signaling pathway.

Taken together, our results suggest that LAITOR is very precise in identifying abstracts of scientific literature mentioning interactions between genes and proteins. LAITOR is able to extract very variable types of protein co-occurrences, no matter how they have been cited in the abstract. In our future work, we intend to adapt LAITOR components to an on-line tool, in which users, as well as computers (using the web services technology) will be able to load their desired literature and perform a LAITOR-based co-occurrence analysis that, integrated with other databases (for example, KEGG [40]), will provide a flexible framework for literature mining-based knowledge discovery.

## Availability and requirements

LAITOR is distributed under the General Public License (GPL). Access http://laitor.sourceforge.net to obtain LAITOR's repository and its documentation from SourceForge.net.

LAITOR requires Linux as operating system, PHP version 5.3.2 or superior, MySQL version 5.0.45 or superior to run. Additional information is found on-line in the LAITOR documentation file.

## Abbreviations

PMID: PubMed Identifier; IAS: Interaction Article Subtask.

## Authors' contributions

ABS created the main idea of the article. ILFM and TGS helped in the development and initial discussion of LAITOR algorithm. ABS and ILFM developed the prototype scripts. ABS developed the final scripts. TGS and RS provided the biointeraction dictionaries. GAP idealized the graph outputs. ABS performed the evaluation and application experiments. JFF and MAAN idealized the concept search and helped in the evaluation experiment. ABS wrote the article. JMO, MANN and RS corrected the article. JMO and RS supervised the initial development of LAITOR. JMO and MAAN supervised the final development of LAITOR. All authors read and approved the final version of the article.

## Supplementary Material

Additional file 1 Application dataset. Click here for file

Additional file 2 Table S1: Example of a protein term and its synonyms representation in the Protein Dictionary. Click here for file

Additional file 3 Plant protein dictionary. Click here for file

Additional file 4 Concepts dictionary. Click here for file

Additional file 5 Table S2: Example of a biointeraction term represented in the Biointeraction Dictionary. Click here for file

Additional file 6 LAITOR co-occurrence pipeline. Click here for file

Additional file 7 Performance evaluation dataset. Click here for file

Additional file 8 Figure S1: Example of a tagged phrase output. Click here for file

Additional file 9 Table S3: Top-10 biointeraction terms most cited in the green plants application analysis. Click here for file

Additional file 10 Figure S2: Full network created by LAITOR from application dataset. Click here for file

Additional file 11 Figure S3: Co-occurrence sub-networks generated by LAITOR. Click here for file

Additional file 12 Table S4: Top-10 concepts terms mostly cited in the co-occurrence analysis. Click here for file

Additional file 13 Figure S4: Hypothesis generation supported by LAITOR output. Click here for file
