# Supplementary material for: LAITOR - Literature Assistant for Identification of Terms co-Occurrences and Relationships
Source: BMC Bioinformatics. 2010 Feb 1;11:70. doi: 10.1186/1471-2105-11-70 (PMC3098111; doi:10.1186/1471-2105-11-70)
Supplement: Additional file 2 — Table S1: Example of a protein term and its synonyms representation in the Protein Dictionary. [file 1471-2105-11-70-S2.DOC]

## Table S1 – Example of a protein term and its synonyms representation in the Protein Dictionary.

| **Name** | **Synonyms** |
| --- | --- |
| PR1 | PATHOGENESIS-RELATED GENE 1 |
| PR1 | PATHOGENESIS-RELATED PROTEIN 1 |
| PR1 | PR-1 |
| PR1 | T6B13.15 |
| PR1 | T6B13_15 |
