# Supplementary material for: LAITOR - Literature Assistant for Identification of Terms co-Occurrences and Relationships
Source: BMC Bioinformatics. 2010 Feb 1;11:70. doi: 10.1186/1471-2105-11-70 (PMC3098111; doi:10.1186/1471-2105-11-70)
Supplement: Additional file 5 — Table S2: Example of a biointeraction term represented in the Biointeraction Dictionary. [file 1471-2105-11-70-S5.DOC]

## Table S2 – Example of a biointeraction term represented in the Biointeraction Dictionary.

| **Name** | **Synonyms** |
| --- | --- |
| TRIGGER | TRIGGER |
| TRIGGER | TRIGGERS |
| TRIGGER | TRIGGERING |
| TRIGGER | TRIGGERED |
