# Supplementary material for: LAITOR - Literature Assistant for Identification of Terms co-Occurrences and Relationships
Source: BMC Bioinformatics. 2010 Feb 1;11:70. doi: 10.1186/1471-2105-11-70 (PMC3098111; doi:10.1186/1471-2105-11-70)
Supplement: Additional file 6 — LAITOR co-occurrence pipeline. [file 1471-2105-11-70-S6.PPT]

## Slide 1
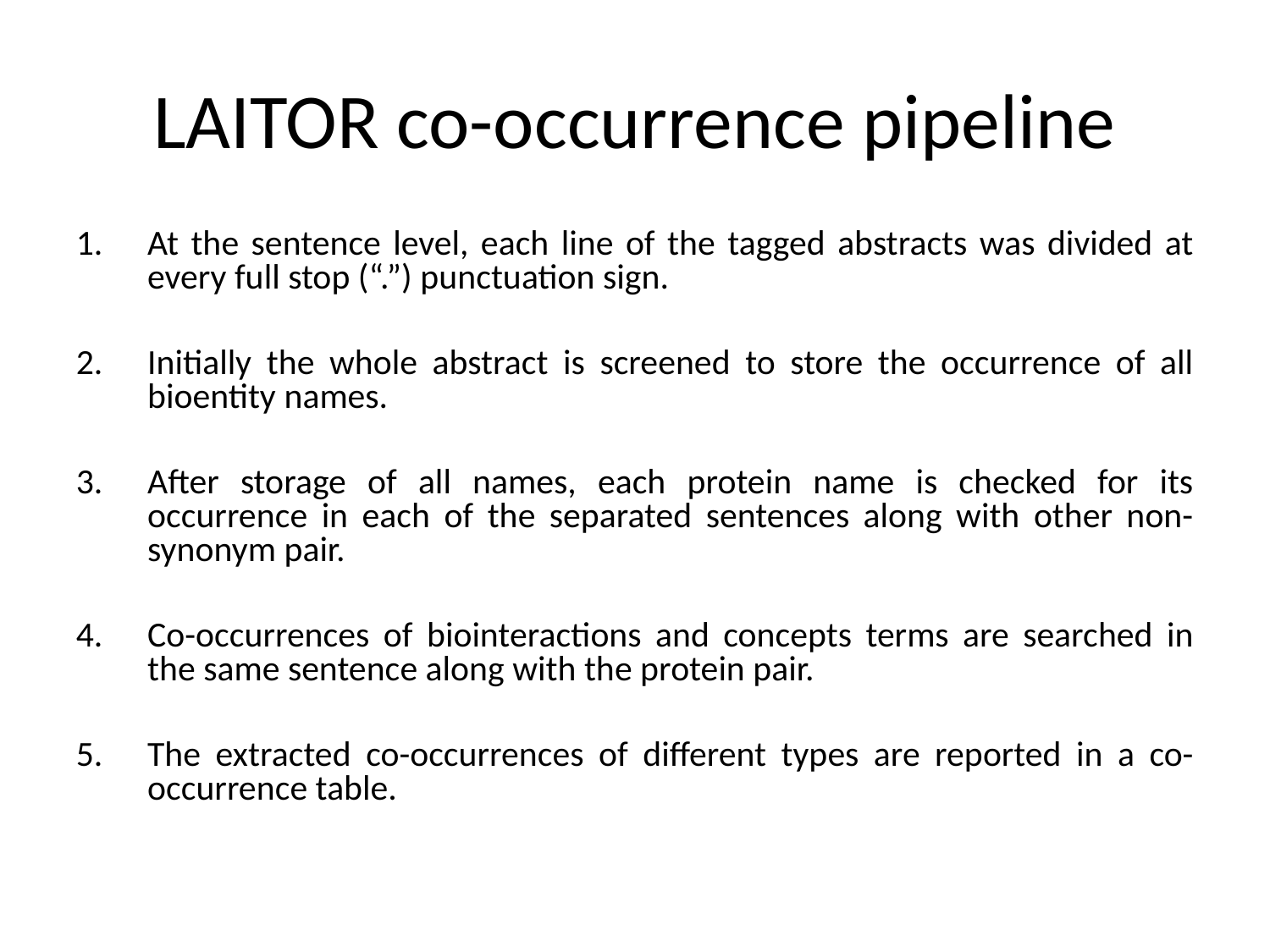

# LAITOR co-occurrence pipeline
At the sentence level, each line of the tagged abstracts was divided at every full stop (“.”) punctuation sign.
Initially the whole abstract is screened to store the occurrence of all bioentity names.
After storage of all names, each protein name is checked for its occurrence in each of the separated sentences along with other non-synonym pair.
Co-occurrences of biointeractions and concepts terms are searched in the same sentence along with the protein pair.
The extracted co-occurrences of different types are reported in a co-occurrence table.

## Slide 2
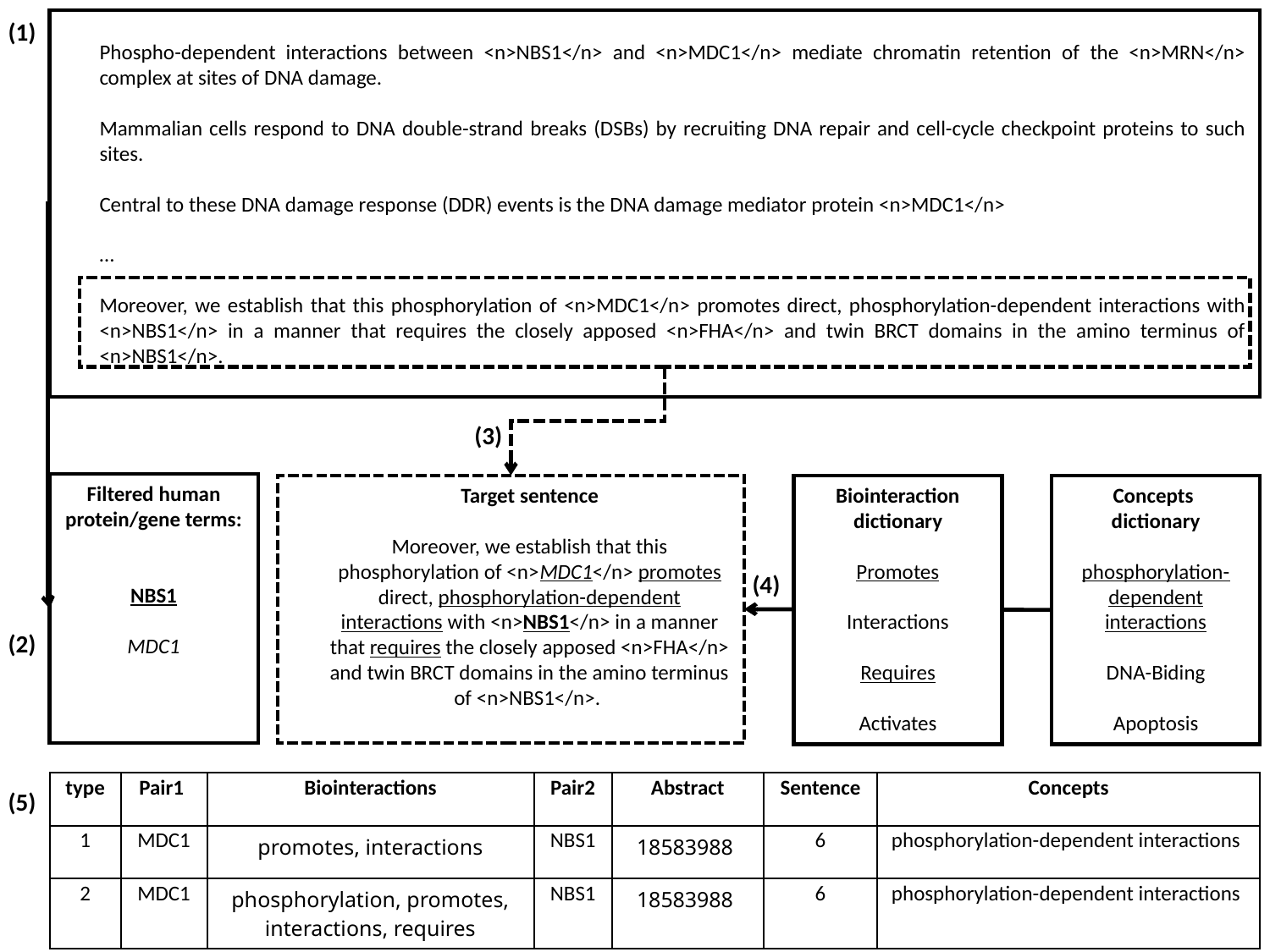

(1)
Phospho-dependent interactions between <n>NBS1</n> and <n>MDC1</n> mediate chromatin retention of the <n>MRN</n> complex at sites of DNA damage.
Mammalian cells respond to DNA double-strand breaks (DSBs) by recruiting DNA repair and cell-cycle checkpoint proteins to such sites.
Central to these DNA damage response (DDR) events is the DNA damage mediator protein <n>MDC1</n>
…
Moreover, we establish that this phosphorylation of <n>MDC1</n> promotes direct, phosphorylation-dependent interactions with <n>NBS1</n> in a manner that requires the closely apposed <n>FHA</n> and twin BRCT domains in the amino terminus of <n>NBS1</n>.
(3)
Filtered human protein/gene terms:
NBS1
MDC1
Target sentence
Moreover, we establish that this phosphorylation of <n>MDC1</n> promotes direct, phosphorylation-dependent interactions with <n>NBS1</n> in a manner that requires the closely apposed <n>FHA</n> and twin BRCT domains in the amino terminus of <n>NBS1</n>.
Biointeraction dictionary
Promotes
Interactions
Requires
Activates
Concepts
dictionary
phosphorylation-dependent interactions
DNA-Biding
Apoptosis
(4)
(2)
| type | Pair1 | Biointeractions | Pair2 | Abstract | Sentence | Concepts |
| --- | --- | --- | --- | --- | --- | --- |
| 1 | MDC1 | promotes, interactions | NBS1 | 18583988 | 6 | phosphorylation-dependent interactions |
| 2 | MDC1 | phosphorylation, promotes, interactions, requires | NBS1 | 18583988 | 6 | phosphorylation-dependent interactions |
(5)
