# Supplementary figures and images for: LAITOR - Literature Assistant for Identification of Terms co-Occurrences and Relationships
Source: BMC Bioinformatics. 2010 Feb 1;11:70. doi: 10.1186/1471-2105-11-70 (PMC3098111; doi:10.1186/1471-2105-11-70)

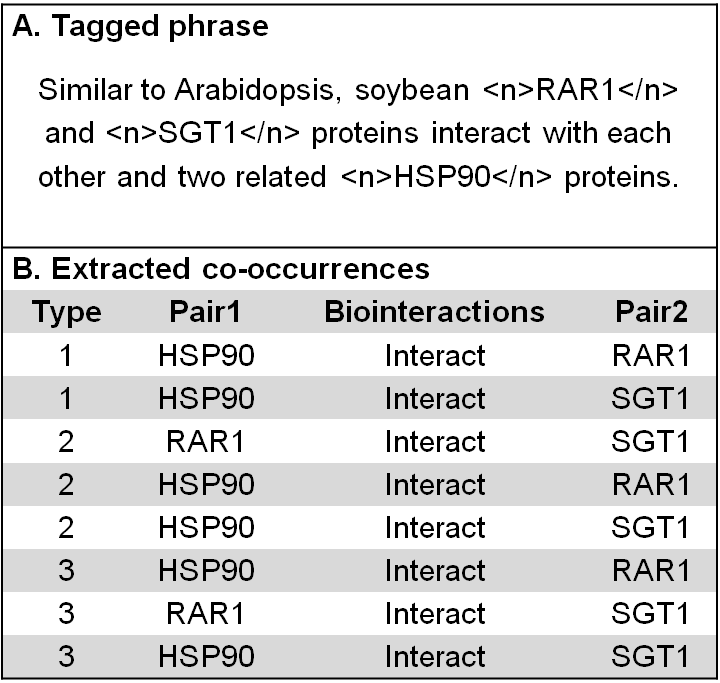

Supplement: Additional file 8 — Figure S1: Example of a tagged phrase output. [file 1471-2105-11-70-S8.TIFF]

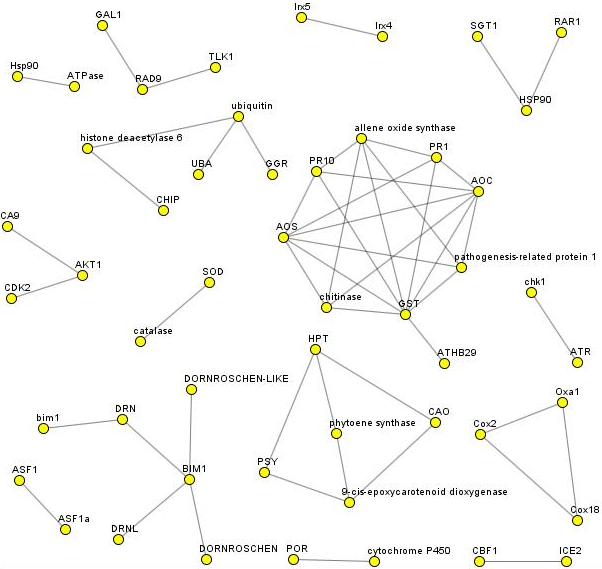

Supplement: Additional file 10 — Figure S2: Full network created by LAITOR from application dataset. [file 1471-2105-11-70-S10.TIFF]

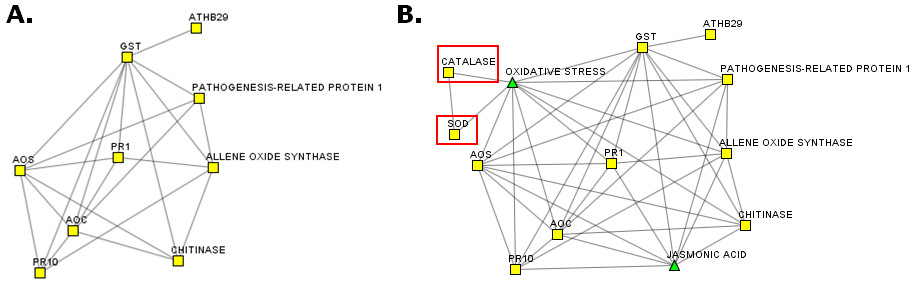

Supplement: Additional file 11 — Figure S3: Co-occurrence sub-networks generated by LAITOR. [file 1471-2105-11-70-S11.TIFF]

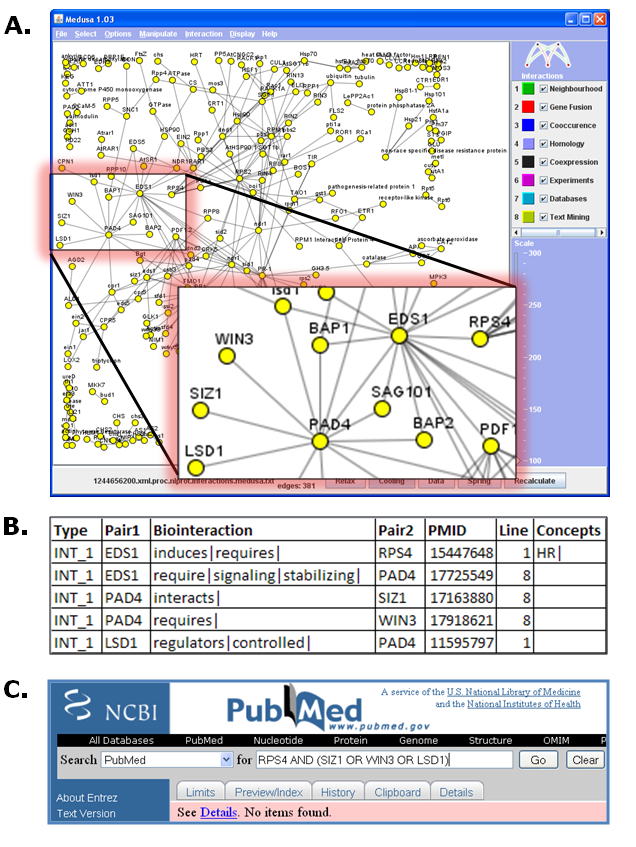

Supplement: Additional file 13 — Figure S4: Hypothesis generation supported by LAITOR output. [file 1471-2105-11-70-S13.TIFF]
