# Supplementary material for: LAITOR - Literature Assistant for Identification of Terms co-Occurrences and Relationships
Source: BMC Bioinformatics. 2010 Feb 1;11:70. doi: 10.1186/1471-2105-11-70 (PMC3098111; doi:10.1186/1471-2105-11-70)
Supplement: Additional file 9 — Table S3: Top-10 biointeraction terms most cited in the green plants application analysis. [file 1471-2105-11-70-S9.DOC]

## Table S3 – Top-10 biointeraction terms most cited in the green plants application analysis.

| **Biointeraction** | **Frequency** |
| --- | --- |
| Encoding | 62 |
| Oxide | 52 |
| Activity | 36 |
| Triggering | 26 |
| Interaction | 18 |
| Expression | 16 |
| Promoter | 16 |
| Induced | 12 |
| Required | 8 |
| Interact | 7 |
