# Supplementary material for: LAITOR - Literature Assistant for Identification of Terms co-Occurrences and Relationships
Source: BMC Bioinformatics. 2010 Feb 1;11:70. doi: 10.1186/1471-2105-11-70 (PMC3098111; doi:10.1186/1471-2105-11-70)
Supplement: Additional file 12 — Table S4: Top-10 concepts terms mostly cited in the co-occurrence analysis. [file 1471-2105-11-70-S12.DOC]

## Table S4 – Top-10 concepts terms mostly cited in the co-occurrence analysis.

| **Concepts** | **Frequency** |
| --- | --- |
| OXIDATIVE STRESS | 27 |
| JASMONIC ACID | 26 |
| ROS | 6 |
| BRASSINOSTEROID | 6 |
| SAR | 5 |
| SALICYLIC ACID | 4 |
| BR | 4 |
| HYPERSENSITIVE RESPONSE | 2 |
| COLD | 1 |
| FREEZING | 1 |

ROS: reactive oxygen species, BR: brassinosteroid, SAR: systemic acquired resistance.
